# How readable are our Patient Information Sheets?

**DOI:** 10.1186/cc14653

**Published:** 2015-03-16

**Authors:** L Strachan, M Booth

**Affiliations:** 1 Glasgow Royal Infirmary, Glasgow, UK

## Introduction

We often need to obtain consent for clinical studies in the ICU. Participant Information Sheets (PIS) can be difficult to understand. A recent French publication [1] supports our hypothesis that PIS have poor readability scores.

## Methods

Protocols submitted for ethics approval between 2008 and 2009 were obtained with permission from the Scotland A Ethics Research Committee. Ethical approval was not required for this observational study. All header, footers, diagrams and tables were removed. Readability scoring was performed using the Flesch Reading Ease and Flesch-Kincaid (FK) grades. Statistical analysis using Excel and MiniTab was then performed. The readability of these documents was compared with everyday documents - newspaper articles, politicians' speeches [2] and standard contract agreements.

## Results

A total of 104 protocols containing 209 PIS were reviewed. Of these, 99 (47%) were written for patients, 56 (27%) for GPs, 26 (12%) for relatives, 17 (8%) for carers, five (2%) for legal representatives and six (3%) were summary sheets only. Sixty-seven (64%) of these protocols were submitted by academic institutions (for example, university or health boards) and 37 (36%) by pharmaceutical companies. Results are expressed as the median and 25th and 75th percentiles. The word count and number of pages were higher for those PIS submitted by pharmaceutical companies compared with academic institutions: 1,561 (471; 5,167) versus 1,177 (626.5; 1,559.8) with *P *< 0.05 and 4 (2; 10) versus 3 (2; 4) with *P *< 0.05 respectively. The Flesch Reading Ease (63 (56; 69) vs. 60 (52.6; 65.4)) and FK grades (3 (5.4; 7.2) vs. 6.8 (6; 7.6)) were similar for both groups. Further subanalysis demonstrated that PIS designed for GPs had a lower word count, lower Flesch and higher FK grade compared with those for patients - the difference in Flesch and FK grade were compared using a Mann-Whitney test and were statistically significant.

## Conclusion

The FK grade is equivalent to US school grade level. The US government advises all policies produced should have a FK grade of <9.Our study suggests that protocols submitted to the ethics committee are easy to read, comparing favourably with broadsheet journalism and standard contract, for example loan contract. However, the average reading age in the UK is 9 years [3], suggesting participants may struggle with the information provided.
